# Expression Profile of Sphingosine Kinase 1 Isoforms in Human Cancer Tissues and Cells: Importance and Clinical Relevance of the Neglected 1b-Isoform

**DOI:** 10.1155/2022/2250407

**Published:** 2022-12-07

**Authors:** Hongjie Chen, Nahal Haddadi, Xiaofeng Zhu, Diana Hatoum, Size Chen, Najah T. Nassif, Yiguang Lin, Eileen M. McGowan

**Affiliations:** ^1^Department of Traditional Chinese Medicine, Third Affiliated Hospital of Sun Yat-sen University, Guangzhou, China; ^2^School of Life Sciences, University of Technology Sydney, Broadway, NSW, Australia; ^3^Department of Transplant Surgery, First Affiliated Hospital of Sun Yat-sen University, Guangzhou, China; ^4^Public Health and College of Arts and Sciences, Phoenicia University, Daoudiye, Lebanon; ^5^Central Laboratory, First Affiliated Hospital of Guangdong Pharmaceutical University, Guangzhou, China; ^6^Guangdong Provincial Engineering Research Center for Esophageal Cancer Precision Therapy, Guangdong Pharmaceutical University, Guangzhou, China

## Abstract

**Background:**

Overexpression of sphingosine kinase 1 (SphK1) is casually associated with many types of cancer, and inhibitors of SphK1 sensitize tumors to chemotherapy. SphK1 is expressed as two major isoforms, SphK1a and SphK1b. To date, no information has been reported on the SphK1 isoform expression profile and its clinical relevance.

**Objective:**

The objective is to examine the expression profile of the SphK1a and SPhK1b isoforms in human cancer and noncancer tissues and cell lines and explore their clinical relevance.

**Methods:**

We used PCR to qualitatively examine the expression profile of these two isoforms in breast, liver, and prostate cancer tissues plus paired adjacent tissues and in 11 cancer and normal cell lines (breast, cervical, bone, prostate, colon, brain, mesothelioma tumor and benign, and human kidney cells).

**Results:**

We found that SphK1a was ubiquitously expressed in all cancer cells and tissues tested; in contrast, SphK1b was only expressed in selective cell types in breast, prostate, and lung cancer.

**Conclusions:**

Our data suggest that SphK1a is important for generic SphK1/S1P functions, and SphK1b mediates specialized and/or unique pathways in a specific type of tissue and could be a biomarker for cancer. This discovery is important for future SphK1-related cancer research and may have clinical implications in drug development associated with SphK1-directed cancer treatment.

## 1. Introduction

The sphingosine kinase 1 (SphK1) isoforms are bioactive lipid enzymes involved in the phosphorylation of sphingosine to produce the active form sphingosine-1-phosphate (S1P), thereby regulating the balance between S1P, sphingosine, and ceramide [1–3]. Maintaining the balance of SphK/S1P signaling is important in normal cellular and physiological processes including cell proliferation, survival, cell death, adhesion, angiogenesis, migration, and inflammation, and it is key in the prevention and progression of cancer [4–14].

Overexpression of SphK1 is linked to many cancer types, including prostate, colorectal, brain, breast, liver, mesothelioma, and other lung diseases [12, 15–21] and has been identified as oncogenic due to “gain of function” in preference to any identifiable mutations [22]. In other words, cancer cells have become reliant on SphK1 expression for survival, a phenomenon termed “non-oncogenic” addiction [22]. Hormone-responsive cancers have been shown to be particularly sensitive to SphK1 expression. Estrogen has been shown to induce SphK1 in both hormone-responsive breast cell lines [23, 24], clinical patient breast cancer samples [25], and triple-negative cell lines [26] and confer tamoxifen resistance [25–27]. The endocrine response can be restored by inhibiting SphK [25, 28]. Similarly, high SphK1 expression is associated with prostate cancer chemotherapy resistance [29], and sensitivity to hormone-resistant prostate cancer cells can be restored by inhibiting the SphK pathway [29–31].

There are two major human SphK1 isoforms, SphK1a (SphK1a-isoform 3, SphK1-43 kDa-GenBank accession: NM_001142601; UniProt ID: Q9NYA1-1) and SphK1b (SK1b-isoform 2, SphK1-51 kDa-GenBank accession: NM_182965; UniProt ID: Q9NYA-2) [32]. Both isoforms share common amino acid sequences, with the exception that the SphK1b isoform has a unique extra 86 amino acid sequence at the N-terminus [32]. This unique N-terminal domain alters the conformation of the SphK1b isoform, allowing for common and additional distinct interaction patterns of SphK1a and SphK1b [33]. Our knowledge of alternative splicing of the SphK1 isoenzyme on cell function is very limited, and many aspects of the functions of the 2 isoforms have not been explored. The first SphK1 isoform described was SphK1a (43 kDa), or isoform 3, and most *in vitro* human SphK1 functional studies have focused on this isoform [8]. SphK1a and SphK1b share the same catalytic domain [34], and no significant differences in S1P activity have been observed [33]. Moreover, there are no overt phenotypic differences in cell morphology or cell proliferation when either SphK1a or SphK1b is stably overexpressed. However, there is little doubt that the expression of one or both isoforms has the capacity to alter downstream cell signaling events [33].

There is some evidence to suggest that changes in the SphK1a/SphK1b ratios affect drug treatment regimens and alter the vulnerability to hormone cancer treatment. In breast cancer cells, specific protein interactions with either SphK1b or SphK1a isoform alter SphK1 signaling events [33]. In prostate cancer cells, alteration in the expression of SphK1a and SphK1b is associated with chemotherapy resistance [35]. Changes in SphK1a and SphK1b levels result in specific changes in ceramide and S1P levels leading to induced apoptosis of androgen-sensitive, but not androgen-independent, LNCaP prostate cancer cells [35]. These studies indicate that the imbalance of SphK1a and SphK1b may be causally associated with cancer progression and resistance to chemotherapy, dependent on hormonal status [35, 36].

We have previously shown that the expression of the SphK1a and SphK1b isoforms in a target cell determines the nature and magnitude of the response to SphK-S1P signaling and can initiate similar and distinct downstream signaling pathways that alter cellular events [33]. For example, SphK1b preferentially interacts with dipeptidyl peptidase 2 (DPP2), a protein involved in the regulation of glucose metabolism [37]. Treatment of hormone-dependent breast cancer cells with a DPP2 inhibitor increases SphK1b expression but does not have an effect on SphK1a expression [33].

Most previous *in vivo* studies so far have explored overall SphK1 expression and activity, not individual isoforms. Historically, most *in vitro* studies use the overexpression of the SphK1a isoform to explore the function of SphK1, and SphK1b is relatively neglected. Differential isoform expression is proving to be emerging as an important hallmark of cancer definition [38]. Individual isoforms of a protein may be differentially expressed in different cancer types or individual cancer cells [39], which may provide adaptive resistance to targeted therapy [40], and we and others have shown the subtleties of differential functional effects of SphK1 isoform expression in cell studies *in vitro* [33, 35, 36]. The exogenous expression status of SphK1b is emerging as having an important impact on signaling pathways [33] and potential interference in hormonal cancer therapy, as shown in prostate cancer cell lines [35, 36]. No specific study thus far has been conducted to explore the expression profile of SphK1a and SphK1b in different cancer cells and tissues. Here, we examined the expression profile of endogenous SphK1a and SphK1b in cancer tissue samples and matched noncancer tissues from the 3 most common types of cancer patients (liver, breast, and prostate cancer) and complemented these studies by examining SphK1a and SphK1b expression in a variety of human cancer and noncancerous cell lines. It is expected that the study will advance our understanding of the functional significance of relative SphK1a and SphK1b in normal and cancer cells and the clinical relevance of these isoforms.

## 2. Materials and Methods

The materials and methods are described in brief. A detailed description can be found in the thesis by Haddadi, 2019 [41].

### 2.1. Cell Culture

Cancer cells were routinely cultured in either Dulbecco's modified eagle medium (DMEM) or Roswell Park Memorial Institute medium (RPMI1640) with 10% fetal bovine serum (FBS) depending on the cell type. Alpha-minimum essential medium (*α*-MEM) supplemented with 2 mM L-glutamine, 100 U/ml penicillin/streptomycin, 20% FCS, 10 ng/ml recombinant basic fibroblastic growth factor was used for primary cell lines. The Lonza MycoAlert™ Plus mycoplasma detection kit was routinely used to check mycoplasma negatively.

### 2.2. MCF-7SphK1 Isoform Expressing Control Cell Lines

MCF-7 breast cancer cells (ATCC : HTB-22™) were used to stably overexpress SphK1a (43 kDa) and SphK1b (51 kDa) containing an N-terminal FLAG-TAG previously described [33, 42]. The SphK1a and SphK1b isoforms were derived from human umbilical vein endothelial cells (HUVECs) [33, 42]. SphK1a and SphK1b cDNA clones expressed the SphK1 isoform 3, variant 3 (NM_001142601), and SphK1 isoform 2, variant 2 (NM_182965) [32].

### 2.3. Cell Lines

Details of the cell lines used for the detection of SphK1a and Sphk1b isoforms are listed in Table 1. Mesothelioma cell lines were kindly donated by Dr. Glen Reid (Asbestos Disease Research Institute–ADRI, Concord, Sydney) and Drs Rayleen Bowman, Walter Berger, and Walter Klepetko, as indicated. Prostate cancer cell lines were kindly donated by the late Robert Sutherland (Garvan Institute of Medical Research, Sydney).

### 2.4. Collection of Clinical Cancer Tissue Samples

Human breast and prostate cancer tissues and adjacent matching tissues were collected from the Third Affiliated Hospital of Sun Yat-sen University. Human liver cancer, hepatocellular carcinoma (HCC) tissues, as well as adjacent tissues from the cancer site, were from the First Affiliated Hospital of Sun Yat-sen University. The use of human tissues for this project was approved by an institutional approval, human ethics GZSCHE 2016-00122.

### 2.5. Western Blots

Cells were lysed in cell lysis buffer for the isolation of whole cell proteins, and protein levels were estimated by using the BioRad BCA assay (Biorad Lab Inc. CA) as previously described [43, 44]. Equal amounts of protein were separated by SDS-PAGE and transferred to polyvinylidene difluoride (PVDF) membranes. The overexpression of the isoforms in MCF-7SphK1a and MCF-7SphK1b was verified by western blot analysis using anti-Flag m2 mouse F1804-1MG from Sigma-Aldrich.

### 2.6. RNA Extraction from Cell Lines

Total RNA was isolated from cultured cells by using either (1) TRIzol (Life Technologies) extraction and RNeasy Plus Mini system (Qiagen) or (2) the Maxwell ESC simply RNA cell system (Promega). Cultured cells were harvested and processed according to the manufacturer's instructions. In all cases, the quality and quantity of RNA were determined by using agarose gel electrophoresis and nanodrop absorbance measurements.

### 2.7. RNA Extraction from Clinical Tissue Samples

Fresh samples of breast, prostate, and HCC cancer tissues and adjacent noncancer tissues were stored in RNA later. RNA extraction was performed by using TRIzol TM according to the manufacturer's instructions. Total RNA purity and concentrations were measured by using a spectrophotometer at wavelength 280 nm.

### 2.8. Reverse Transcription Polymerase Chain Reaction (PCR)

Reverse transcription (RT) was performed by using the SuperScript III First-Strand Synthesis SuperMix (Life Technologies) with oligo (dT) priming, according to the manufacturer's protocol. RT-PCR reactions were optimized and conducted by using the Eppendorf master cycler gradient instrument.

### 2.9. Optimisation of the SphK1-PCR Primers

SphK1-specific primers were designed to pair in different combinations (Supplementary Figure 1) with locations as shown on the SphK1 sequence (accession number NM_182965.2) (Supplementary Figure 1A). The expected sizes of the SphK1-PCR amplified products, alongside the SphK1 sequence locations, are listed in Supplementary Figure 1B. Each of the 6 primer sets was tested to determine the optimal PCR conditions for each primer set by using cDNA derived from the MCF-7-SphK1b and SphK1a stably transfected cells.

## 3. Results

### 3.1. Differential Expression of SphK1a and SphK1b Isoform Is Cell Line Dependent

**Figure 1:**
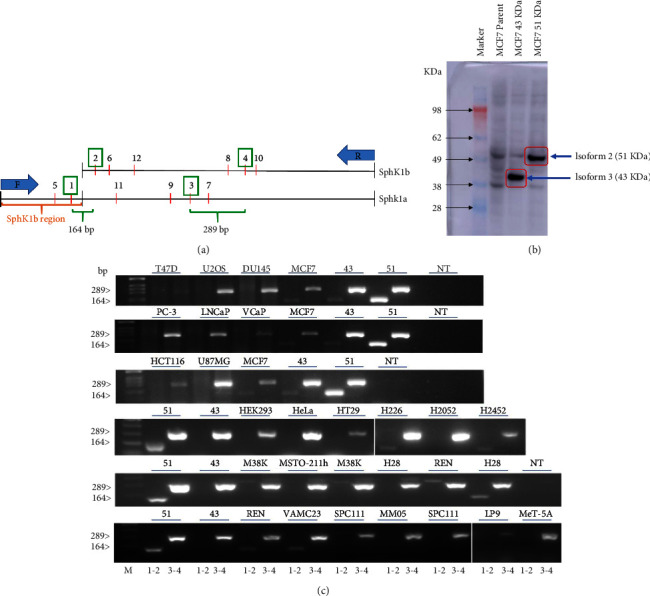
Differential expression of SphK1a and 1b isoforms in cancer cells *in vitro*. (a) Schematic of the SphK1a and 1b primers (F: forward; R: reverse) locations (reference Supplementary Figure 1, primer sequences, and SphK1 sequence locations). (b) Western blot visualization of SphK1a (isoform 3, 43 kDa) and SphK1b (isoform 2, 51 kDa) in stably transfected MCF7 cells detected by using a flag-tag antibody [33]. (c) Representative gels of RT-PCR amplification products of SphK1 isoforms from cancer and noncancer cell lines (described in Tables 1 and 2). RT-PCR was performed by using SphK1 primers F1-R2 [1, 2] and F3-R4 [3, 4]. Primers F1-R2 amplified a product of 164 bp in length (overlapping the SphK1a-b N′ terminal region) and primers F3-R4 amplified a product of 289 bp (within the SphK1a region). MCF-7SphK1b [45] and MCF-7SphK1a [43] and no DNA template (NT) were used as controls for every set of RT-PCR reactions. These panels are representative of repeat experiments.

In the absence of antibodies to clearly define the true nature of endogenous SphK1a (43 kDa) and SphK1b (51 kDa) protein expression by using western blot, we chose to develop a simple, economical, easy-to-use, differential SphK1a and SphK1b RT-PCR technique. Using this SphK1-RT-PCR diagnostic test, we can determine unique SphK1b expression in any cell and tissue type. We designed and evaluated a number of specific SphK1-PCR primers, forward (F) and reverse (R) (Supplementary Figures 1 and 1A). The N' terminal region of the SphK1b-a gene is a high G-C-rich area and contains a number of secondary hairpin loop structures, making the upstream “SphK1b-a” region difficult to amplify. After careful testing, we chose a unique 164 bp (base pair) PCR primer set (F1, R2), which overlaps the SphK1a-1b N' terminal region, thus identifying cells containing only SphK1b and the 289 bp PCR primer set (F3, R4), within the SphK1a region picking up both SphK1 isoforms. These two PCR primer sets (F1-R2) and (F3-R4) were proved to be the most robust and consistent for routine testing of SphK1 isoform products in both cell lines and patient tissue samples. Cells stably overexpressing SphK1a and SphK1b manufactured previously [33] were used as controls to test the effectiveness of PCR primers and the efficiency of amplification of SphK1-PCR products. The overexpression of MCF-7 (SphK1a-43 kDa; isoform (3) and MCF-7SphK1b-51 kDa; isoform (2)) was confirmed by using western blot (Figure 1(b)). MCF-7 cells have very low but detectable levels of endogenous SphK1a and 1b. We tested different cancer cell types for SphK1a and 1b expression, namely, breast, prostate, colon, brain, bone, ovarian, and mesothelioma (epithelioid and biphasic), as well as benign mesothelioma and human embryonic kidney (HEK) (24 cell lines), as listed in Supplementary Table 1. All cell lines, irrespective of cell type, showed the 289 bp PCR corresponding to a region within the SphK1a domain (Figure 1(b)). In contrast, the 164 bp PCR product, which is unique for the SphK1b isoform, was cell line specific. SphK1b was only detected in selective breast and mesothelioma cell lines. The unique SphK1b primers (F1-R2) did not detect a product in prostate cancer (androgen dependence and independence), colon, ovarian, brain, and bone cell lines. Of the 2 breast cancer cell lines tested, only MCF-7 cells detected SphK1b expression. Interestingly, the majority of the mesothelioma epithelioid cell lines expressed detectable SphK1b (5/6) as well as 2/3 benign mesothelioma cells. The 3 biphasic mesothelioma cell lines tested did not express detectable SphK1b products. These results are summarized in Table 1.

### 3.2. Expression of SphK1a Is Ubiquitous in Different Human Tissue Types and SphK1b Isoform Is Tissue-Type Dependent

**Figure 2:**
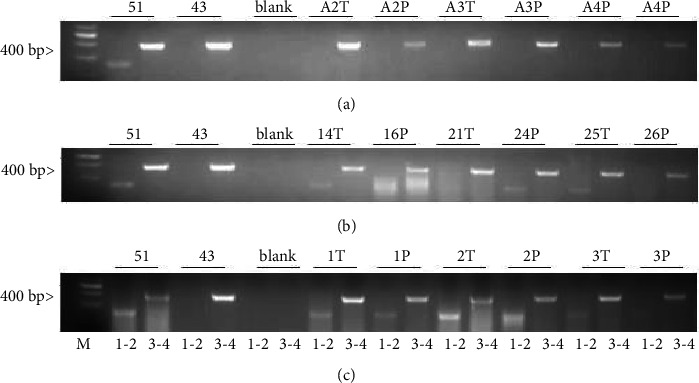
Comparative analysis of SphK1a and SphK1b isoform expression in human cancer and adjacent tissues. Representative gels of RT-PCR amplification products of SphK1 isoforms from human cancer and adjacent tissue samples. RT-PCR was performed by using SphK1 primers F1-R2 and F3-R4. Primers F1-R2 amplified a product of 164 bp unique to the SphK1b isoform and primers F3-R4 amplified a product of 289 bp within the SphK1a region. (a) Liver, (b) prostate, and (c) breast cancer and adjacent tissues. MCF-7SphK1b [45] and MCF-7SphK1a [43] and no DNA template (NT) were used as controls; T: tumor; *P*: adjacent tissue. Each sample was amplified 2x with similar results.

Resections from 6 patients diagnosed with HCC, 7 patients diagnosed with prostate cancer, and 15 patients with breast cancer, along with matching adjacent tissues, were analysed for expression of SphK1a and SphK1b isoforms by using RT-PCR. Patient details, diagnosis information, age of diagnosis, subtype, gender, and hormonal status, where appropriate, alongside SphK1 isoform expression analysis, are listed in Suppl.  Table 2. Visual representations of the SphK1a and SphK1b PCR amplified products are shown in Figure 2.

The SphK1a isoform was expressed in all liver cancer tissues and the corresponding adjacent tissues; in contrast, the SphK1b isoform was not detected in any of the liver cancer tissues or the corresponding adjacent tissues (Table 2 and Figure 2(a)).

All human prostate samples, both cancer and adjacent tissue, expressed the SphK1a isoform (Table 2 and Figure 2(b)). The SphK1b isoform was detected in 71% (5/7) prostate cancer tissues and 57% (4/7) corresponding adjacent tissues; there was no demarcation in SphK1 isoform expression to distinguish tumor stage (Table 2 and Figure 2(b)). In contrast, no endogenous expression of SphK1b was observed in any of the prostate cancer cell lines tested *in vitro,* as shown in Table 1 and Figure 1.

There appeared to be some discrepancies in the detection of SphK1a and SphK1b in breast tissue. In breast tissue, 93% (14/15) of breast cancer samples and 66% (10/15) of corresponding adjacent tissue expressed detectable levels of SphK1a (Figure 2(c) and Table 2). Detection of the unique SphK1b isoform was 60% (9/15) and adjacent 53% (8/15) breast cancer patient samples. When SphK1 isoform expression status was considered by breast cancer grade (Table 3) and by hormonal status (ER+ or ER−) (Table 4), we found that most of Grade 1, 2, IDC, and ILC tissue resections had undetectable levels of SphK1b isoform, whereas all Grade 3 breast tissues proved positive for SphK1b expression (Table 4). Hormone receptor-positive breast cancers were more likely to express both SphK1 isoforms.

Profiling SphK1 isoform expression of patient tissue samples shows that SphK1a is detected in the liver, prostate, and breast, both in cancer and adjacent tissues. However, there are some discrepancies in individual breast samples where SphK1 is not detected. These results are consistent with the findings that all cells tested *in vitro*, independent of cell type, detected a product within the common SphK1a. Expression of SphK1b was found to be cell type-specific; it was not detected in the liver and was not expressed in all prostate and breast tissues tested (Table 2 and Figures 2(a)–2(c)). Again, this is consistent with cell line profiling, where SphK1b is not universally expressed in all cell lines and cell types.

In summary, all human tissues tested were positive for SphK1 independent of tissue type, and although this RT-PCR assay is not quantitative, there was no clear difference in SphK1 expression between cancer and adjacent tissues. The SphK1b isoform was not detected in any sample of human liver cancer and noncancer liver tissues. SphK1b was detected in over 64% of human prostates and 57% of breast tissues tested suggesting a functional role for SphK1b in reproductive tissues. Given that breast cancer cells and prostate cancer cells have shown different drug responses depending on SphK1a or SphK1b expression, this may have some relevance in potential drug therapy [33, 36, 46, 47].

### 3.3. Comparative Stability of SphK1a and SphK1b RNA Structure

**Figure 3:**
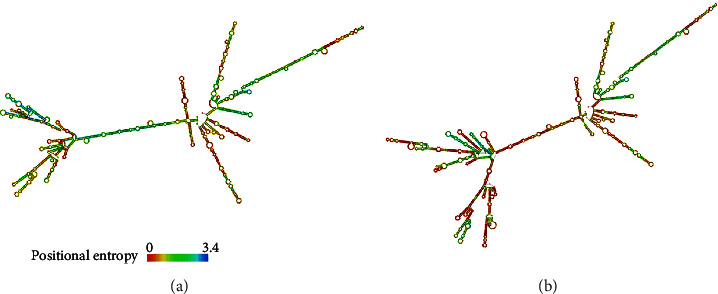
RNA secondary structure prediction by minimum free energy that may have higher fidelity of the predicted structures for SphK1a (a) and SphK1b (b) isoforms. The positional entropy with low entropy is predicted with high confidence. The mRNA secondary structure fold predictions were performed based on highly probable base pairs and the lowest free energy structure for each sequence as determined by RNAfold WebServer on the ViennaRNA web services (http://rna.tbi.univie.ac.at/cgi-bin/RNAWebSuite/RNAfold.cgi).

Given that in all experiments SphK1b was less abundantly expressed than SphK1a, we compared the folding of mRNA and the secondary structures formed by the 2 isoforms to examine the stability of the two RNA structures. Using computational modelling predictions [48], the b-isoform RNA was predicted to be more unstable than the shorter SphK1a isoform (Figure 3), consistent with our previous findings, where the SphK1a protein isoform was found to be more stably expressed [33]. This prediction is based on the concept that mRNAs associated with stress have higher free energy, longer loop length, and more single strands that enable them to undergo conformational changes in response to their environment [49]. Moreover, some mRNA isoforms transcribed from a single gene can have different half-lives depending on their environmental conditions [50, 51]. Investigations by Geisberg and his group, examining stabilizing and destabilizing elements in mRNAs and isoform half-lives, suggested that double-stranded structures at the 3 “region” are crucial in mRNA stability [45]. Taking all these predictions into account, SphK1b with the higher free energy and the longer loop length is predicted to be less stable than the SphK1a isoform.

## 4. Discussion

There is substantial evidence to show that SphK1 plays a critical role in many types of cancer progression, as well as many other chronic diseases [13, 14, 52–55]. For example, in estrogen-responsive breast cancer cells, elevated SphK1 is associated with endocrine resistance [24, 28], and the spatial organization of SphK1 in ER positive breast tumors is aligned with the prognostic outcome [56]. Hence, the strong motivation to find SphK inhibitors to combat drug resistance in cancer patients [53].

**Figure 4:**
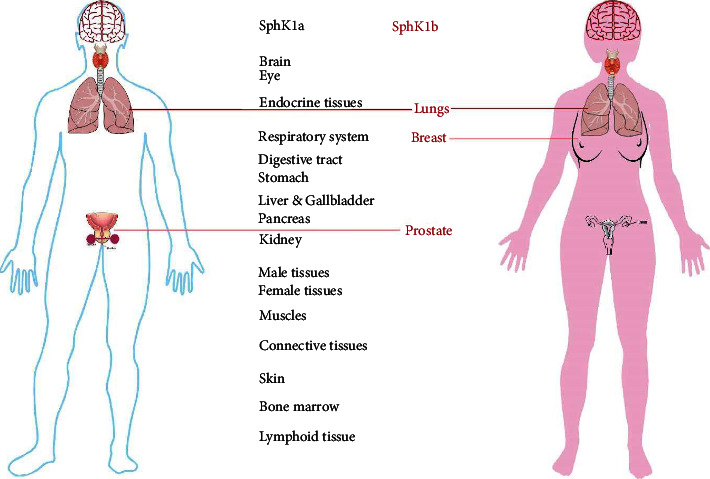
Distribution of SphK-1a and -1b isoforms in human tissue. The SphK1 isoenzyme is expressed in all human organs (https://www.proteinatlas.org/ENSG00000176170-SPHK1/tissue). SphK1 is expressed as 2 major isoforms (SphK-1a and -1b). Data on individual isoform expression are limited. Our knowledge to date suggests that SphK1a is ubiquitously expressed in all tissues, where the expression of SphK1b has only been reported in lung, breast, and prostate tissues.

The SphK1 isoenzyme is expressed in most tissues (Figure 4), and its role as a major player in development and disease has been widely researched over the past 20 years, including its role in cancers, diabetes, and liver pathology [9, 13, 14, 27, 52, 53, 57, 58]. The relevance and consequence of individual SphK1 isoform expression, especially the longer SphK1b isoform, in cancer cells are underexplored and unclear. Characterization of individual isoforms has been elusive due to their common and compensatory functions, and most SphK-S1P research has focused on the SphK1 and SphK2 isoenzymes. Conventionally, the shorter human SphK1a isoform has been studied in most *in vitro* cell studies [59], and the SphK1b isoform has been neglected.

SphK1a and 1b isoforms have distinct as well as compensatory cellular signaling pathways; however, we know little about their definitive expression in normal and cancer human tissues. The little we do know is mainly derived from the few *in vitro* studies where the individual isoform is overexpressed and may have significance in altering SphK1 function, both in normal physiology and in cancer pathophysiology.

### 4.1. Consequences of SphK1b Expression in Cell Lines

In the last 12 years, there have been some emerging *in vitro* studies obtained from prostate and breast cancer cell lines to suggest that differential expression of SphK1a and SphK1b can affect some cell functions and, more importantly, may play a critical role in drug efficacy. In 2010, the Pyne group published a seminal paper on the importance and subtleties of the role of SphK1a and SphK1b expression in cell lines with reference to the anti-SphK (SKi) action on cell apoptosis [36]. They first established that the SKi inhibitor-induced proteasomal degradation of SphK1a in human pulmonary aortic smooth muscle cells (hPASMCs), MCF-7 breast cancer cells, and androgen-sensitive prostate cancer cells was associated with the onset of apoptosis. However, interestingly, in androgen-independent prostate cancer cells, SKi failed to induce apoptosis but induced cell cycle arrest, and this was linked to SphK1b expression; SphK1b expression in androgen-independent cells was found to be associated with increased resistance to SKi-induced proteasomal degradation preventing apoptosis. When forced inhibition of SphK1 was performed, using a siRNA targeted to the common region of SphK1a and SphK1b in combination with SKi, apoptosis was induced, and this was associated with an increase in ubiquitin-proteasomal degradation of SK1 [36]. Additional research in 2012 [35] further demonstrated that the two isoforms exhibited different properties in the cell, and SphK1b resistance to SKi proteasomal degradation, in part, is defined by SphK1b expression levels and regulatory properties unique to SphK1b. Reduced sensitivity to apoptosis associated with SKi/SphK1b in androgen-independent prostate cells was also aligned with specific changes in ceramide and S1P levels [35]. These results suggest that properties unique to the N-terminal 86 amino acids of the 1b isoform confer additional mechanisms to protect cells from apoptosis and block the cell cycle in the G0 phase, which are dependent on the cell type and different cell properties.

In our previous study, we characterized the expression of SphK1a and SphK1b, respectively, in MCF-7 cells and investigated the protein-protein interactions of the two isoforms. Proteomic studies by our group demonstrated common and discrete interacting partners for both isoforms in an estrogen receptor-positive breast cancer cell line [33]. The unique SphK1b-86 kDa upstream region provides subtle and not-so-subtle differences in protein interactions and downstream signaling pathways in MCF-7 cells, with the potential to change drug response [33]. Although SphK1b and SphK1a common and specific interacting partners were identified, there were no overt differences in cell proliferation, cell morphology, and SphK1-S1P activity between cells expressing the different isoforms. However, differences in the localization of the SphK1a and Sphk1b proteins were observed; SphK1b was predominantly cytoplasmic, and SphK1a was located in the cytoplasm and nucleus. Localization of these 2 isoforms, in part, as well as the unique upstream 86 amino acids of the SphK1b, may add to different interacting partners and different functions in the cell. As mentioned, the localization of SphK1 has been identified as a marker of breast cancer prognosis. Of particular interest is that one of the top unique SphK1b interacting partners was DPP2. DPP2, among other functions, has been identified as an essential survival factor in quiescent cells and an essential protein in the maintenance of the G0-phase of the cell cycle; cells expressing DPP2 do not undergo apoptosis but remain in the state of quiescence [60]. Deletion of DPP2 has been shown, in some cell lines, to promote apoptosis [60]. Therefore, speculatively, the binding of SphK1b to DPP2 may be one mechanism involved in the prevention of apoptosis but not cell cycle arrest. Inhibition of DPP2, reducing the SphK1b-DPP2 interaction, results in SphK1b increase. It would be interesting to see whether blocking the interaction of SphK1b-DPP2 interaction not only increases SphK1b but also increases SphK1b sensitively to SKi ubiquitin-proteasomal degradation and affects apoptosis.

### 4.2. Differential Expression of SphK1a and 1b in Different Cell Types and Clinical Samples

The main objective of this study was to determine whether both endogenous SphK1a and SphK1b are expressed in human tissue by using a simple PCR test and whether loss or gain of one or both isoforms could be linked to cancer.

This study demonstrates that the SphK-1b isoform is selectively expressed depending on the tissue/cell type. Alternatively, SphK1a is ubiquitously and dominantly expressed in all cell lines and human tissue types tested, implying that SphK1a is important for generic SphK1/S1P functions and maybe the principal isoform involved in mediating the known prosurvival and cell maintenance functions of SphK1 [8].

SphK1 has been identified as a novel target for mesothelioma [20, 61], and here we show that benign and mesothelioma epithelioid cell lines express detectable levels of SphK1b, whereas only SphK1a is detectable in biphasic mesothelioma cell lines. The significance of these findings has not yet been defined. Conversely, the selective cell and tissue expression of SphK1b in breast and prostate, based on the limited *in vitro* experiments, suggests that this isoform mediates specialized and/or unique pathways, i.e., the 1b-isoform tissue specificity is important in regulating or modulating signaling pathways for specific cell functions.

There is little, if any, information available on the endogenous expression of SphK isoforms in human tissues, and most of the *in vitro* human SphK1 functional studies have either focused on the SphK1a shorter isoform or not specified which isoform is being studied and reviewed in [8, 32]. *In vitro* studies focusing on the expression of the two main SphK1 isoforms (1a and 1b) suggest that an imbalance or aberrant expression of these isoforms plays a role in subverting the signaling pathways involved in resistance to treatment. In particular, SphK1b isoform expression may influence treatment outcomes in tumors of the reproductive glands, including prostate [35, 36, 47] and breast [33]. The few *in vitro* experiments mentioned demonstrated that SphK1b expression reduces drug sensitivity in hormone-responsive prostate cancer cells, suggesting that the expression of SphK1a and 1b in prostate cancer patients may be significant in the selection of treatment with the potential to desensitize hormone treatment response [33, 35, 36].

In breast cancer cell lines, endogenous SphK1b and SphK1a are present in low amounts in MCF-7 cells. However, SphK1b was not observed in T-47D cells, which are less responsive to estrogen. SphK1a and SphK1b isoforms were present in all grade 3 breast cancers and adjacent tissues, whereas the 1b isoform was not detected in the majority of grade 1 and 2 breast cancers. When we subdivided the breast tissue samples into ER positive and ER negative status, ER positive breast cancers were more likely to express both SphK1 isoforms (Table 4). Overexpression of SphK1 has been shown to be a mediator of estrogen signaling and is causally associated with endocrine resistance in ER+ breast cancer cells, and silencing of SphK1 activity can restore sensitivity [24, 28]. Our understanding of the association between SphK1 expression in normal and malignant breast cells [12, 27] and this added knowledge that SphK-1a and -1b are expressed in estrogen-responsive normal and breast cancer tissue provides another avenue to explore anti-SphK novel targeted therapeutic intervention.

The findings in this study, that the 1b-isoform is expressed in breast and prostate tissue, support further experimentation into determining how alterations in SphK isoforms may affect drug responsiveness, drug resistance, and interaction with other noncancer treatments [33, 35, 36, 62]. As previously mentioned, one such interaction was the preferential binding of SphK1b to DPP. In response to the addition of a DPP2/4 inhibitor SphK1b expression increased significantly, but not SphK1a [33], DPP4 is currently used as a drug to manage type II diabetes mellitus [62, 63] and is also associated with inflammatory control [62]. As diabetic patients have a greater risk of developing cancer and patients with comorbidity (cancer and diabetes) have poorer outcomes [9], the expression of SphK1b may influence the prognosis and treatment of the patient.

We demonstrated that SphK1a is expressed in all cancer cells and tissue types tested, and expression of the 1b-isoform is cell- and tissue-specific; the human prostate, breast, and lung expressed both SphK1 isoforms; however, liver tissues (cancer and adjacent) only expressed the SphK1a isoform. SphK1 has been revealed as a critical regulator in liver disease [64] and given that SphK1b was not detected in resected human liver samples, suggesting that the SphK1a isoform is critical for liver function, and the 1b-isoform is redundant. In contrast, selective tissue expression of SphK1b (breast and prostate) suggests that the 1b isoform is important for specialized cell functions in different tissues, supported by *in vitro* evidence. As referred to the limited published *in vitro* information available, the expression of SphK1b may negatively affect treatment outcomes in some breast and prostate cancers.

Although one of our objectives was to determine if there was a demarcation between SphK1a and SphK1b expression, comparing cancer tissues and adjacent tissues, in this study, we did not find any clear distinction between the expression of the two isoforms in cancer tissues and adjacent tissues. Albeit, knowing that both isoforms are expressed in reproductive tissue (normal and cancer tissue), this suggests that individual isoforms are important in normal physiological as well as pathophysiological. Although we have some evidence to show SphK1a and -1b can influence signaling pathways, understanding if, or how, SphK1b activity modulates hormone activity broaches a new pathway of discovery. Potentially, from a cancer prognostic viewpoint, this information will aid our understanding of how SphK1 isoform expression may affect treatment outcomes, especially in patients with hormone-responsive cancers.

In both in cell lines (*in vitro*) and in human tissues (*in vivo*), when expressed, the SphK1b isoform was consistently less abundant compared with the SphK1a isoform, given the limitation that this assay was qualitative and not quantitative. When we compared the mRNA folding and secondary structures formed by the 2 isoforms, using computational modelling predictions [48], the b-isoform was predicted to be more unstable than the shorter SphK1a isoform (Figure 3). These predictions, although implied, may provide one reason as to why the SphK1b isoform seems of lesser abundance compared with its shorter 1a isoform, *in vitro* (cell lines) and *in vivo* (patient tissue samples), i.e., SphK1b RNA may be more unstable and/or susceptible to degradation within the cell milieu.

In our previous study, at the protein level, the unique C-terminal 86 amino acids of SphK1b allow conformational changes to facilitate preferential isoform interactions with proteasomal proteins and ubiquitin-protein ligases [33]. Therefore, subjectively, at both the mRNA and protein level, the longer SphK1b isoform may be more unstable and preferentially more susceptible to degradation control. However, some *in vitro* studies in prostate cancer cell lines favour SphK1b as being more stable than the SphK1a isoform [35, 36]. Again, these seemingly opposing findings may be dependent on the cell milieu or cell type dependent.

Our previous study demonstrating the important differences between SphK1a and SphK1b as drivers of distinct and in-common signaling pathways [33] provides some insight into the divergence of 1a- and 1b-isoform regulation of cell signaling pathways and functions. At this stage, we have no direct evidence to suggest the association of the SphK1 isoform with chemoresistance or hormone resistance in cancer patients. Nonetheless, aberrant SphK1 isoform expression has been causally associated with prostate cancer therapy resistance in preclinical laboratory experiments. Although SphK1 inhibitors have been successful in increasing chemosensitivity [29, 65, 66], there are examples demonstrating discriminatory chemosensitivity depending on the expression of the two major SphK1 isoforms in hormone-dependent and independent prostate cancer cell lines [35, 36]. Similarly, differences in SphK1a and SphK1b expression had different functional responses in breast cancer cells [33].

The limitations of our study include that the assessment of SphK1 isoform expression is based only on data from qualitative PCR rather than quantitative PCR. Furthermore, the sample size of the patients is relatively small, limiting the generalization of the findings.

## 5. Conclusions

This is the first report specifically examining the expression profile of SphK1a and SphK1b in 3 common human cancer tissues (lung, breast, and liver) and 11 human cell types. Our results provide the first insight into the ubiquitous nature of SphK1a expression and selectivity of SphK1b expression in different types of cancer and adjacent tissues, supporting any impact of SphK1b on normal cell function, cancer progression, and/or treatment outcome, is more likely to be cell-tissue type specific. Conceivably, the cell specificity of SphK1b expression may play a significant role in modulating or enhancing SphK1 signaling and physiological functions, especially in hormone-related cancers. SphK1b expression may change cell sensitivity to anti-SphK1 drugs and affect other drug interactions. For example, the SphK1b unique N-terminal interactions are associated with subtle changes in how cells respond to medicinal drugs. Alternatively, SphK1b expression may function as a biomarker for some cancer types. What is emerging is that, as we learn more about the role of individual SphK1 isoform functions, the understanding of SphK1 isoform expression in cancer may become an important factor in personalized designer anti-SphK drug therapy [67].

## Figures and Tables

**Table 1:** Summary of SphK1 isoform expression in different cell line types *(in vitro)*.

Cell type	Cell origin	SphK1a^*∗*^	SphK1b^*∗∗*^
Breast	Epithelial	2/2	1/2
Cervical	Epithelial	1/1	0/1
Bone	Epithelial	1/1	0/1
Prostate	Epithelial	4/4	0/4
Colon	Epithelial	2/2	0/2
Brain	Epithelial	1/1	0/1
Mesothelioma	Epithelioid	6/6	5/6
Mesothelioma	Biphasic	3/3	0/3
Mesothelioma	Benign	3/3	2/3
Human embryonic kidney (HEK)	Epithelial	1/1	0/1

Total		24/24	8/24

^*∗*^SphK1a primers, F3-R4; ^*∗∗*^SphK1b primers, F1-R2. *Note.* Refer to Supplementary Table 1 for full analysis.

**Table 2:** Summary of SphK1 isoform expression in liver, prostate, and breast cancer and nontumor tissue.

Tissue type	SphK1a^*∗*^ (cancer)	SphK1a^*∗*^ (adjacent)	Total	SphK1b^*∗∗*^ (cancer)	SphK1b^*∗∗*^ (adjacent)	Total
Liver	6/6	6/6	12/12	0/6	0/6	0/12
Prostate	7/7	7/7	14/14	5/7	4/7	9/14
Breast	14/15	10/15	24/30	9/15	8/15	17/30

Total	27/28	23/28	50/56	14/28	12/28	26/56

^*∗*^SphK1a primers, F1-R2; ^*∗∗*^SphK1b primers, F3-R4.

**Table 3:** Analysis of SphK1 isoform expression in breast cancer patients by grade.

Grade	Total no.	Cancer		Adjacent	
		SphK1a	SphK1b	SphK1a	SphK1b
Grade 1 + 2	8/15	7/8	2/8	4/8	2/8
Grade 3	6/15	6/6	6/6	6/6	6/6
ILC	1/15	1/1	0/1	0/1	0/1

ILC: invasive lobular carcinoma.

**Table 4:** Analysis of SphK1 isoforms in breast cancer patients by hormonal status.

Type	Total no.	Cancer		Adjacent	
		SphK1a	SphK1b	SphK1a	SphK1b
Breast overall	15	14/15	9/15	10/15	8/15
ER+ (8/15)	8	8/8	6/8	8/8	5/8
ER− (7/15)	7	6/7	3/7	3/7	2/7

## Data Availability

The data used to support the findings of this study are included within the article and the supplementary materials.

## Supplementary Materials

Supplementary Materials Figure S1. SphK1a and SphK1b PCR primer design. A) the SphK1 sequence showing the location of the SphK1 primers. B. SphK1 primer sequences (Forward and reverse). Each primer sequence is numbered, and colour coded which refers to the mapping positioning in Fig. 1A. The expected size of the PCR product is shown. Table S1. SphK1 isoform expression profiles of cancer and non-cancer cell lines.Table S2. Tumour characteristics and SphK1 isoform expression in human cancer and noncancer tissues.
