# Supplementary material for: Expression Profile of Sphingosine Kinase 1 Isoforms in Human Cancer Tissues and Cells: Importance and Clinical Relevance of the Neglected 1b-Isoform
Source: J Oncol. 2022 Dec 7;2022:2250407. doi: 10.1155/2022/2250407 (PMC9750787; doi:10.1155/2022/2250407)
Supplement: Supplementary Materials — Figure S1. SphK1a and SphK1b PCR primer design. A) the SphK1 sequence showing the location of the SphK1 primers. B. SphK1 primer sequences (Forward and reverse). Each primer sequence is numbered, and colour coded which refers to the mapping positioning in Fig. 1A. The expected size of the PCR product is shown. Table S1. SphK1 isoform expression profiles of cancer and non-cancer cell lines.Table S2. Tumour characteristics and SphK1 isoform expression in human cancer and noncancer tissues. [file 2250407.f1.docx]

**Figure S1: SphK1a and SphK1b PCR primer design**


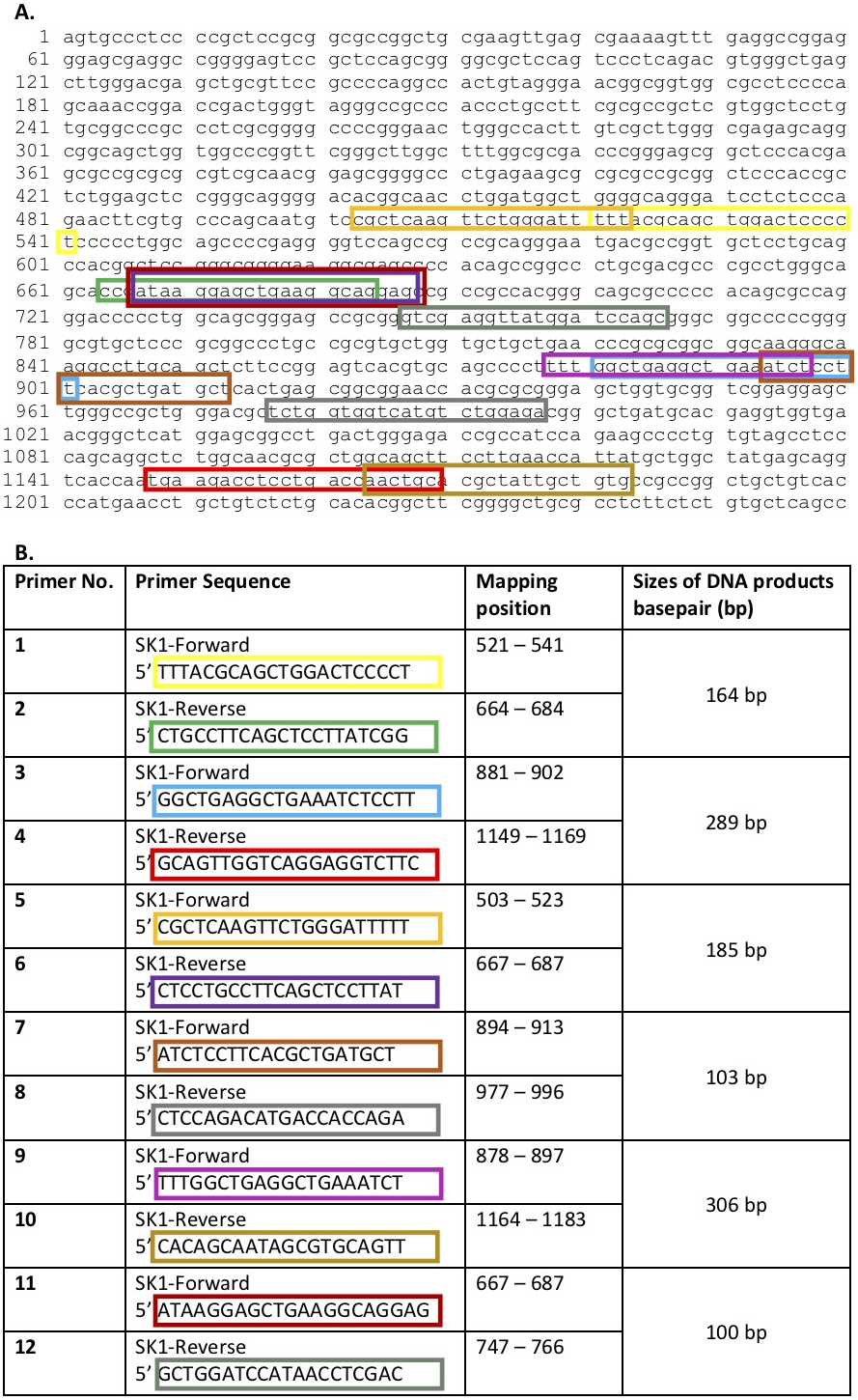


**Figure S1.** SphK1a and SphK1b PCR primer design. A) the SphK1 sequence showing the location of the SphK1 primers. B. SphK1 primer sequences (Forward and reverse). Each primer sequence is numbered, and colour coded which refers to the mapping positioning in Fig. 1A. The expected size of the PCR product is shown.

**Supplementary Tables: SphK1 isoform expression profiles of cancer and non-cancer tissues and cell lines**

**Table S1. SphK1 isoform expression profiles of cancer and non-cancer cell lines.**

| **Cancer type** | **Reference/source** | **(SphK1a: primers F3-R4)** | **(SphK1b: primers F1-R2)** | |
| --- | --- | --- | --- | --- |
| **Breast Cancer (epithelial)** |  |  | |  |
| MCF-7 luminal A (ER+/PR+/HER2-) | ATCC® HTB-22™ | Y | | Y |
| MCF-7-SphK1a |  | Y | | Y |
| MCF-7-SphK1b |  | Y | | Y |
| T-47D luminal A (ER+/PR+/HER2-) | ATCC® HTB-133™ | Y | | N |
| **Cervical cancer** |  |  | |  |
| HeLa | ATCC® CCL-2™ | Y | | N |
| **Bone cancer (Epithelial)** |  |  | |  |
| U-2OS (osteosarcoma) | ATCC® HTB-96™ | Y | | N |
| **Prostate cancer (Epithelial)** |  |  | |  |
| DU 145 (androgen independent) | ATCC® HTB-81™ | Y | | N |
| LNCaP (androgen dependent) | ATCC® CRL-1740™ | Y | | N |
| PC-3 (androgen independent) | ATCC® CRL-1435™ | Y | | N |
| VCaP (androgen independent) | ATCC® CRL-2876™ | Y | | N |
| **Colon cancer (Epithelial)** |  |  | |  |
| HCT 116 | ATCC® CCL-247™ | Y | | N |
| HT29 | ATCC® HTB-38™ | Y | | N |
| **Brain cancer (Epithelial)** |  |  | |  |
| U-87MG | ATCC® HTB-14™ | Y | | N |
| **Mesothelioma (Epithelioid)** |  |  | |  |
| NCL-H28 (*p113)  NCL-H28 (*p114) | ATCC® CRL-5820™ | Y  Y | | N  Y |
| NCL-H226 (*p74)  NCL-H226 (*p75) | ATCC® CRL-5826™ | Y  Y | | Y  Y |
| NCL-H2052 (*p51)  NCL-H2052 (*p89) | ATCC® CRL-5915™ | Y  Y | | Y  N |
| NCL-H2452 (*p36)  NCL-H2452 (*p37) | ATCC® CRL-5946™ | Y  Y | | Y  N |
| MM05 (*p25)  MM05 (*p32) | [38]* | Y  Y | | N  N |
| VAMC23 (*p57) | [39]** | Y | | Y |
| **Mesothelioma (Biphasic)** |  |  | |  |
| MSTO-211h (*p56) | ATCC® CRL-2081™ | Y | | N |
| SPC111 (*p14)  SPC111 (*p16) | [40]** | Y  Y | | N  N |
| M38K P5 (*p5)  M38K P5 (*p6) | [41] | Y  Y | | N  N |
| **Mesothelioma (Benign)** |  |  | |  |
| REN (*p14) | [42] | Y | | Y |
| REN (*p15) |  | Y | | Y |
| MeT-5A (1A) | ATCC® CRL-9444™ | Y | | N |
| MeT-5A (1B) |  | Y | | N |
| LP9 (*p3) | [43] | Y | | Y |
| LP9(*p14) |  | Y | | N |
| **Non-tumorigenic** |  |  | |  |
| Human embryonic kidney (HEK293) | ATCC® CRL-1573™ | Y | | N |

*A/Prof. Rayleen Bowman (UQ Thoracic Research Centre, The Prince Charles Hospital, Brisbane, Australia)

** Prof. Walter Berger (Institute of Cancer Research) & Prof. Walter Klepetko (Division of Thoracic Surgery, Medical University of Vienna, Austria).

*Note:* RNA from the mesothelioma cell lines were kindly provided by Dr Glen Reid and Patrick Winata (ADRI).

Note *p = passage number

Table S2. Tumour characteristics and SphK1 isoform expression in human cancer and noncancer tissues.

| Type | Nature | Age/Sex | subtype | SphK1a | SphK1b |
| --- | --- | --- | --- | --- | --- |
| *Liver |  |  |  |  |  |
| A1T | Cancer | 62/Male | HCC | Y | N |
| A1P | Adjacent |  |  | Y | N |
| A2T | Cancer | 63/M | HCC | Y | N |
| A2P | Adjacent |  |  | Y | N |
| A3T | Cancer | 62/F | HCC | Y | N |
| A3P | Adjacent |  |  | Y | N |
| A4T | Cancer | 66/M | HCC | Y | N |
| A4P | Adjacent |  |  | Y | N |
| A5T | Cancer | 36/M | HCC | Y | N |
| A5P | Adjacent |  |  | Y | N |
| A6T | Cancer | 66/M | HCC | Y | N |
| A6P | Adjacent |  |  | Y | N |
| ***Prostate |  |  |  |  |  |
| 9T | Cancer | 57/M | pT3a stage | Y | Y |
| 10P | Adjacent |  |  | Y | Y |
| 14T,15T | Cancer | 75/M | pT2c stage | Y | Y |
| 16P | Adjacent |  |  | Y | Y |
| 17T,18T | Cancer | 72/M | pT2c stage | Y | Y |
| 19P | Adjacent |  |  | Y | N |
| 21T,22T | Cancer | 70/M | pT3b stage | Y | N |
| 23P,24P | Adjacent |  |  | Y | Y |
| 25T | Cancer | 75/M | pT2a stage | Y | Y |
| 26P | Adjacent |  |  | Y | Y |
| 27T | Cancer | 69/M | pT3b stage | Y | N |
| 28P | Adjacent |  |  | Y | N |
| 29T | Cancer | 64/M | pT3b stage | Y | Y |
| 30P | Adjacent |  |  | Y | N |
| *Breast |  |  |  |  |  |
| 1T | Tumour | 34/F | 30% IDC(Grade:3); 70% DCIS (High Grade)/(ER+、PR-、HER2-) | Y | Y |
| 1P | Adjacent |  |  | Y | Y |
| 2T | Tumour | 42/F | IDC(Grade:3)/(ER-、PR-、HER2+) | Y | Y |
| 2P | Adjacent |  |  | Y | Y |
| 3T | Tumour | 47/F | IDC(Grade:3)/(ER+、PR+、HER2+) | Y | Y |
| 3P | Adjacent |  |  | Y | Y |
| 4T | Tumour | 66/F | 8% IDC(Grade2); 92% DCIS (Medium Grade)/(ER-、PR-、HER2+) | Y | N |
| 4P | Adjacent | 66/F |  | N | N |
| 5T | Tumour | 32/F | IDC (Grade 2)/(ER-、PR+、HER2+) | Y | Y |
| 5P | Adjacent |  |  | N | N |
| 6T | Tumour | 60/F | IDC (Grade 2) | Y | N |
| 6P | Adjacent |  |  | N | N |
| 7T | Tumour | 61/F | IDC (Grade 2)/(ER-、PR+、HER2+) | N | N |
| 7P | Adjacent |  |  | Y | Y |
| 8T | Tumour | 62/F | 10% IDC (Grade 1); 90% DCIS (Low Grade)/(ER+、PR+、HER2+) | Y | Y |
| 8P | Adjacent |  |  | Y | N |
| 9T | Tumour | 47/F | ILC | Y | N |
| 9P | Adjacent |  |  | N | N |
| 10T | Tumour | 60/F | IDC (Grade 2)/(ER+、PR+、HER2+) | Y | N |
| 10P | Adjacent |  |  | Y | Y |
| 11T | Tumour | 76/F | IDC (Grade 2)/(ER+、PR+、HER2+) | Y | N |
| 11P | Adjacent |  |  | N | N |
| 12T | Tumour | 53/F | 90% IDC (Grade 2); 10% DCIS/(ER+、PR+、HER2+) | Y | Y |
| 12P | Adjacent |  |  | Y | N |
| 13T | Tumour | 54/F | IDC (Grade 3)/ER-、PR-、HER2-) | Y | Y |
| 13P | Adjacent |  |  | Y | Y |
| 14T | Tumour | 43/F | IDC (Grade 3)/ER+、PR+、HER2+) | Y | Y |
| 14P | Adjacent |  |  | Y | Y |
| 15T | Tumour | 50/F | IDC (Grade 3)/ER+、PR+、HER2+) | Y | Y |
| 15P | Adjacent |  |  | Y | Y |

Note: HCC – hepatocellular carcinoma.

* Kindly provided by Prof Xiaofeng Zhu

** Kindly provided by Dr Meijun Long,

*** Kindly provided Dr Hongjie Chen.
